# Bioethical Considerations of Advancing the Application of Marine Biotechnology and Aquaculture

**DOI:** 10.3390/md15070197

**Published:** 2017-06-24

**Authors:** Reginal M. Harrell

**Affiliations:** Department of Environmental Science and Technology, University of Maryland, 2113 AnSc/AgEng Bldg, College Park, MD 20742, USA; rharrell@umd.edu

**Keywords:** ethics, bioethics, marine biotechnology, aquaculture, alternative ethical considerations

## Abstract

Normative ethical considerations of growth of the marine biotechnology and aquaculture disciplines in biopharming, food production, and marine products commercialization from a bioethical perspective have been limited. This paucity of information begs the question of what constitutes a bioethical approach (i.e., respect for individuals or autonomy; beneficence, nonmaleficence, and justice) to marine biotechnology and aquaculture, and whether it is one that is appropriate for consideration. Currently, thoughtful discussion on the bioethical implications of use, development, and commercialization of marine organisms or their products, as well as potential environmental effects, defaults to human biomedicine as a model. One must question the validity of using human bioethical principlism moral norms for appropriating a responsible marine biotechnology and aquaculture ethic. When considering potential impacts within these disciplines, deference must be given to differing value systems in order to find common ground to advance knowledge and avoid emotive impasses that can hinder the science and its application. The import of bioethical considerations when conducting research and/or production is discussed. This discussion is directed toward applying bioethical principles toward technology used for food, biomedical development (e.g., biopharming), or as model species for advancement of knowledge for human diseases.

## 1. Introduction

Modern civilization began with the Neolithic Revolution with the transition from a hunter-gatherer to an agrarian society that involved the use of technology [1]. Over the following millennia, technology advanced to meet the increased production needed to support population growth. Food production was increased by applying technologies, including those related to selective breeding of crop plants and animals [2]. Likewise, technologies have evolved to include molecular approaches such as those that yield chimeric organisms or those that lead to removal of genes using targeted “molecular scissors” [3].

Transitioning from traditional breeding and improvement in husbandry practices to genetic manipulation is linked to our expanding ability to incorporate technological advances driven by our scientific curiosity. Technology, therefore, is integral to advancement of humanity as man is a toolmaker, and tools have always been part and parcel to advancement. Granted other animals use tools such as otters using stones to open mollusk shells, but only man “is marked by its whole-scale commitment to develop and use new tools ([4], p. 1)”. Biotechnology then “is a set of technologies specifically aimed at manipulating living things, including human beings themselves, arguably for the common good ([4], p. 1)”. Thus, historically from an applied ethical perspective, biotechnology has a strong utilitarian scope.

Genetic technologies interface with genetic codes, which are the information that is passed from parent to offspring in successive generations and is the basis for all the activities carried out in living organisms. It is the information essential for development, cellular differentiation, instructions for cell function, gene expression, and reproduction; not just within the cell but within the organism itself [5]. Like natural shifts in the genetic code, intentional and artifactual changes in the genetic material in an organism have the possibility to be passed to subsequent generations. The latter often has an associated public perception of risk and potentially altering the “naturalness” of the organism that can cause consternation among a portion of the general public [6,7,8,9,10,11,12,13,14,15,16,17,18,19,20,21,22]. Yet, the public readily consumes food, even organic varieties, or uses plant or animal-derived products that have been effected and affected through the use of genetic selection and genetic manipulation that removes the consumed or applied item far from its original or natural state.

Globally the drivers of marine biotechnology and aquaculture opportunities involve plant and animals for seafood production [23,24,25,26]; use as bioreactors of novel proteins, enzymes, and products that have industrial, nutraceutical, or pharmaceutical uses (e.g., biopharming) [6,7,8,9]; or, serve as a model for human genetics understandings and/or disease treatments [10,27,28]. These are often accomplished by genetic engineering producing transgenic, genetically modified organisms (GMO). In the latter, animals are used as models for experimentation and to develop and evaluate new biotechnological research in determining a product’s potential use or harm to humans before clinical trials begin. Thus, because technological applications are being adopted for use in humans and/or the environment, it is incumbent to separate biotechnological advances of in vitro research for development and application from that of in vivo operations for food production as the former does not have the societal-value constraints found with the latter [11,12,29]. To that end, practical considerations for distinguishing aquaculture from harvesting wild or capture fisheries involve the concept of ownership of the stock and deliberate human intervention in some aspect of the life cycle [30], which lessens the concern from an environmental point of view. 

From an aquaculture perspective, it is clear that the world fisheries are overfished with regard to providing a sustainable production of seafood to meet global demand [23,24,25,26]. Yet, because of population growth this demand for seafood and its products is continuing to increase annually and aquaculture and associated advances in technology are being touted as a means to keep production on pace with demand. For instance, in 2004 more than 240 species, 94 families (including 146 fish, 53 mollusks, 30 crustaceans and 9 plant species) were being produced [24,25]. In 2012, this number increased to over 565 “species” being cultivated with an estimate exceeding 600 species including over 200 species in China alone [26]. In 2009, aquaculture production was ~55.7 mmT, but by 2014 production levels had increased to ~73.8 mmT [26]. It was in 2014 that a milestone was reached wherein the aquaculture’s sector contribution for human seafood consumption exceeded that of wild-caught products [26].

From a societal and bioethical perspective, when science begins manipulating the genomes of animals (and plants to a lesser degree) public concerns arise. Interesting enough, it is not the act of the manipulation of genomes that causes the most angst among society. Instead, it is the public’s perception of the risks of biotechnology. These risks relate to embedded social, cultural, political, and religious values, and how these risks alter individual values and the real or perceived impacts these risks may have on the environment and/or animal welfare or organism integrity [13,31,32,33,34,35,36,37,38]. Second to perception of risk is the issue of public trust—in risk regulation, risk management, institutional responsibility, and the media or public-relations sources to make the correct choices and provide truthful information [14,15,35,36,37,38]. To that end, there may be several issues as to how the public will respond to biotechnology in product development and/or aquaculture that pulls in people different directions [9].

The details of the variety of biotechnological applications associated with the value of marine products and aquaculture are beyond the scope of this paper and the reader is referred to other articles addressed in this journal’s special edition. Normative ethical theory orientations and discussions such as consequential, deontological, virtue, or justice ethics are not the primary focus of this paper either, nor are general applied ethical discussions within agriculture in general and aquaculture specifically. Such discussions, as well as the differences associated with the various ethical worldviews can be found elsewhere (see [6,7,9,10,12,22,33]). The rest of this commentary will specifically address bioethical perspectives and considerations as they relate to marine biotechnology and aquaculture.

## 2. Bioethical Considerations

There is a plethora of information written about bioethics, but, for all intents and purposes, the application is for human medical and end-of-life decisions (see [34] for compendium of information). There are few scholarly works on the bioethics of non-humans, which are restricted to terrestrial agriculture and domestic farm animals as a source of food or for human-health purposes (e.g., xenotransplantation wherein animals are used for human organ transplants [12,22]). Few works are available for aquatic vertebrate organisms and almost none for other flora and fauna as they relate to the bioethics of marine biotechnology. Some of the references provided above link to bioethics in aquaculture. Given this information, it is important to realize that bioethical principles are basically related to biotechnology and biomedicine as they apply to human health and well-being. 

Since we live in a pluralistic, transnational society, what constitutes the ethics behind bioethics is often confusing. In fact, there is some question as to whether bioethics exists at all. Turner ([39] p. 778) explains this question by stating:

“In a sense—if by bioethics is meant a shared mode of normative analysis, a common way of thinking, an overarching framework for moral deliberation, a recognized set of tools with which to reason and debate or a widely accepted ethics decision-making model—does not exist. … Normative reasoning is supposed to lead towards an understanding of how individuals should conduct themselves, how institutions should be designed and organised and how policies can be made intellectually coherent and morally defensible. Within the theoretical and methodological compendium of bioethics, various approaches begin from distinctive premises about human nature, justice, and social organisation and often proceed to different normative conclusions. …, There is no recognizable, widely shared, common moral philosophy that bioethicists draw upon to resolve moral disputes. There is no common creed, widely accepted method of moral reasoning or normative theory, or practical body that binds together bioethicists.”

This quote from Turner demonstrates that, if the study and application of human bioethics has difficulty in developing a consistent standard as to what constitutes a proper and recognized bioethical approaches for use in biomedical fields, one must wonder how it can be translated to a non-human application. 

This question becomes apparent because the “gold standard” by which the field of bioethics was founded, *The Principles of Biomedical Ethics* by Tom Beauchamp and James Childress ([34] and later editions), provides a vocabulary and intellectual framework of principles that are used to address human medical, healthcare, and biotechnology issues only. Their approach is best known as the principlism guidelines for bioethical decision making because it follows an outlined set of rules. Yet today, principlism is just one of many different approaches in bioethics [39]. 

Beginning in the 1980s and continuing into the 1990s, bioethicists separated themselves into two primary camps; “principlists” who emphasize moral principles, and “casuists” who focus on situational ethics wherein decisions are dependent on circumstances and specific cases [39]. A third group, the “real” bioethicists, engage “realistic scientists and concerned biologists and physicians who have an intuition to help build a ‘Bridge to the Future’ whether of (*sic*) not their effort is labeled ‘bioethics’” [40]. These latter individuals see the world as practitioners and not philosophers or ethicists. They blend a mixture of biocentrism with humanism and focus on the “needs, interests, and welfare of human beings”; in essence, an anthropocentrism that relates to the ‘common good’ of the human species [40]. Due to the rigidity of “real bioethicists” focusing on humans only, discussion of their tenants to marine biotechnology and aquaculture would be speculation.

The diversity of bioethical approaches demonstrates that plurality of cultures, faiths, attitudes, and beliefs has a tremendous influence on one’s ethical perspective as it is one of the components that shapes our worldview. Thus, the resultant multiple theoretical and methodological approaches in bioethics demonstrates that the advocates, assumptions, and modes of argumentation impact our normative and hence bioethical conclusions [39]. 

The ensuing discussion will focus on the precepts of principlism as it is the accepted standard and will heavily depend on Beauchamp and Childress’ [34] seminal work. Casuistry will not be discussed in this commentary as it will serve only to confuse the issue as it can become problematic due to its case by case evaluative component. As such it is somewhat relativistic, which can lead to expressing moralistic fallacies that become a slippery-slope fallacy in itself. As marine biotechnology and aquaculture are still in their “moralistic infancy”, it is better to start with accepted principles before we begin to build a code of bioethical approaches to these non-human disciplines.

### 2.1. Moral Status

Before we begin a discussion on bioethical principles, we must understand if the organism under study or production is a moral subject whose welfare or welfare interests must be considered [41]. If we should consider its welfare or welfare interests, then it has moral standing. To have moral standing means the subject warrants moral consideration [34,41]. At the heart of bioethics is the task of answering the question who or what has moral standing. The moral agent (i.e., the scientist, aquaculturist, or biotechnologist) who is manipulating the subject is the one who has moral responsibility and can act morally or immorally, and who can be judged to have acted incorrectly on moral grounds [41].

The first question of whether the subjects of marine biotechnological advances or aquaculture operations are moral subjects or not must be addressed. Unfortunately, deciding if a fish, shrimp, mollusk, or other invertebrate such as horseshoe crabs, squid, or coral polyps are moral subjects is not obvious. It depends, in part, on which normative ethic one uses to evaluate the question as each ethic (consequentialism, deontological, virtue, etc.) has differing perspectives and can shape answers differently. Dependent on individual ethical values, the answers as to whether an organism’s welfare or welfare interests are the topic of consideration may well depend on if the animal is sentient (i.e., can feel pain) [42]. In addition, it may need to be decided not only if it is sentient but does it have higher cognitive functions that allow it to decide what that pain means to it as an entity expressed as a positive or negative stimulus [43]. From a finfish perspective, to address the moral status question in more detail, Bovenkerk and Meijboom [17,18] provide an excellent overview, and the reader is referred to their discussion for a better understanding of the complexity of the issue. For purposes of the rest of this discussion, I will make the broad assumption that live organisms (not component parts) subjected to the realm of marine biotechnology and/or aquaculture are moral subjects worthy of moral status and consideration.

### 2.2. Moral Norms

Within the principlism approach, there are four main moral norms to consider as to whether an action is bioethical or not; autonomy, non-maleficence, beneficence, and justice [34]. As these are the standards by which principlists derive their decisions, including answering the question of moral considerability, there is a significant amount of refereed publications that reference these principles. Thus, they will be only explained in the broadest of context. A brief discussion of each principle definition will follow with respect to its application within marine biotechnology and/or aquaculture.

### 2.3. Autonomy

The moral norm of autonomy is integral to principlism; albeit it does not override all other moral considerations, nor is it necessarily individualistic, focused on reason, or unduly legalistic [34]. While a precise definition is disputed, functionally it is the capacity to act with the ability to choose and act on those choices for oneself (self-rule) without compulsion, influence by others, some external force, and from certain limitations such as inadequate understanding that prevents meaningful choice [34,41]. In other words, an autonomous individual acts freely and in accordance to a self-chosen plan [34]. 

Clearly autonomy is a human construct as humans, to our current level of understanding, are the only animal that can meet these particular criteria; especially the components of cognitive understanding and meaningful choices. While this is somewhat disputed with studies of the higher primates and certain species such as dolphins under confinement choice experiments, it is not clear if those animals are responding to cognitive reasoning, or they are expressing learned responses [42,43,44].

Where then does this leave us with organisms used in marine biotechnology such as lower vertebrates, invertebrates, plants, and embryos? Even if the animal—organism—is classified or categorized as sentient and does have the cognitive ability to make such informed choices (therein meeting two main normative ethical theoretical constructs of consequentialism and deontology), our limitations in understanding how those choices may be communicated are still a major stumbling block to be able to honor an autonomous choice. If we are to honor the moral norm of autonomy, then how are we to proceed? 

As the moral agent responsible for the welfare of the moral subject in question we have four significant choices. First, we can ignore concerns about their welfare. Second, we can act on the subject’s behalf as an individual. Third, we can assume a defined role as a surrogate on their behalf. Lastly, we can apply a uniform code of ethics that has been determined by a cross-section of scientists, ethicists, regulators, and the general public. The fourth option is specifically designed to consider the balance of advancing technology and providing safe food or food-product choices that are effected while prima fasciae considering the welfare and/or welfare interests of the moral subjects at hand. 

The first choice is untenable as it is morally irresponsible as there are no checks and balances that would prohibit gratuitous evil (a moral concern) from occurring. Here profit is probably the main consideration and the costs of responsible and moral welfare operation of the science or business could be relegated to a secondary priority.

The second choice places an individual or organization in an uncomfortable position similar to the first option because the checks and balances are of concern; albeit not at the same level as doing nothing. In this option, one must be aware of not only the moral subject’s physical and emotional well-being, they must be aware of the impact the technology or organism (i.e., GMO) may have on environmental risks as well, as it is a violation of public-trust issues. In addition, when acting on behalf of a moral subject one again has a prima fasciae responsibility to protect the moral subject from negligence and harm. The individual also has to avoid the appearance of or actual conflict of interest to the overall operation of the business as the scope of meaning of protection may supersede the business responsibilities to the owners, stockholders, and other employees. 

In human autonomy considerations, an example of the third choice may be a system paralleling a court-appointed surrogate such as one would find caring for a child (i.e., Guardian ad litem). Here, the legal constraints of what can and cannot be done in these cases has yet, to my knowledge, to be tested in non-human organisms. Under these conditions, there would be precedents that must be adhered to and appropriately reported under the watch-care of a court or organizational-appointed authority. From a human perspective, a qualified surrogate must have: (1) the ability to make reasoned judgments, (2) adequate knowledge and information, (3) emotional stability, and (4) a commitment to an incompetent’s patient’s interests, free of conflicts of interest, free of controlling influence by those who might not act in the patient’s best interest [34]. As appropriate, it might be advantageous to modify these criteria for anyone who is to act as a surrogate for non-human organisms subjected to marine biotechnological research and/or aquaculture production.

The fourth option appears to be best and is in force in many aquaculture operations and associations globally. For instance, the World Food and Agriculture Organization has a code of ethics in fisheries, as does the Federation of European Aquaculture Producers, and the U.S. Department of Commerce with its Code of Conduct for Aquaculture Development in the U.S. Exclusive Economic Zone. While not perfect, these codes are thoughtfully and carefully developed. They offer responsible and acceptable standards for all aspects of aquaculture from research, production, marketing and retailing. They also consider the impact on the cultured organism(s) and the environment in which they are reared or into which they may potentially escape. Lastly, they factor in impacts to the consumer.

### 2.4. Nonmaleficence

This moral norm imposes an obligation not to inflict harm on others. In medical ethics, it is closely linked to the maxim *Primum non nocere*: “Above all do no harm” [34]. From an institutional perspective, this principle is contained within the practice of the three “Rs”; Refinement, Reduction, and Replacement. Being self-explanatory, doing no harm is almost impossible when conducting biotechnological research or aquaculture production as the moral subjects are manipulated, have genes spliced or inserted, crowded, subjected to stress, and, even under certain circumstances, necessarily euthanized. At best, from a food-production perspective as found in aquaculture, humane slaughter is as essential in fish production and harvest as it is in livestock or poultry operations where the animal is humanely and quickly euthanized and processed for human consumption.

For aquaculture production purposes, replacement or reduction are the opposite of operational goals. Therefore, it is incumbent on owners and operators to minimize any factor that may cause undue discomfort or duress to the animals being produced. To a producer this statement appears to be common sense as they know that any stressor impacts growth and feed conversion performance, which increases input costs, and lowers output profits. As such, they try to maximize the well-being of the organism being cultured. However, to the uninitiated public, misunderstandings and perceptions of ill-treatment and crowding can impact their willingness to purchase farm-reared animals. This reluctance is present regardless of whether the organism is cultured indoors, outdoors, or in the wild. 

To the researcher, while laws and/or regulations of animal care and use are not extended to non-vertebrates, care should still be taken to consider if a non-animal model can be used in place of the species in question. Likewise, scientists have the responsibility to conduct statistical power analyses to determine the optimal number of organisms needed to conduct proper experiments and not to use more organisms than necessary. Last, from the researcher and the producer perspective, any opportunity to refine the research or production protocols wherein the moral subject suffers as little as possible is essential. Minimizing pain and suffering at any phase of research and/or production is critical to improving the welfare of the organism, whether you consider the animal sentient or not. To effect this protocol, you must have an understanding of the optimal conditions in which the organism lives within its natural environment.

Like the principle of autonomy, the moral agent’s ability to understand all the nuanced implications of research or production on their subject’s well-being is at task. When a researcher-producer is the voice of the moral subject, ideally, he/she must understand and comprehend, to the best of their ability, the implications all aspects of the research or production protocols may have on the well-being of the subject. Yet, one wonders if this is asking too much of a highly-specialized scientist or technologist. 

The interdisciplinary nature of this science rarely considers a priori the socio-political and ethical implications of the work, so is it fair to expect complete understanding of the encompassing specifics of nonmaleficence? Can any one individual such as a principal investigator truly be expected to know all the implications of their team’s research? Probably not. That is why science has become interdisciplinary. However, we still think in a box. In today’s world of society and politics driving the funding for science we cannot ignore the ethical implications of our research, especially when it is as complex as biotechnology that may have implications for what we eat, how we are healed, and its potential impact to our environment and non-human inhabitants. The ethical implications of “Above all do no harm” cannot be ignored either. This is exactly why the biomedical profession has ethical review boards and academic institutions have ethicists on their Animal Care and Use Committees and their Institutional Review Boards.

Thus, the limitations of such efforts include a lack of a common language and an insufficient or inadequate understanding of the driving theory behind the differing perspectives. Such limitations may become a hindrance and risk to advancement of the science because of developing frustrations between two well-intentioned partners. A possible answer to address this and other bioethical concerns is to integrate an ethicist (especially one who is literate in the sciences in question) from the conceptual to the completion stages of the science. Including ethical considerations as a form of “checks and balances” for societal acceptance may serve to quickly advance the developing technology in an appropriate and embraceable manner. 

### 2.5. Beneficence

Linked directly to nonmaleficence is the moral norm of beneficence, which is the opposite of nonmaleficence. In the former you do no harm, in the latter you take positive action to contribute to the subject’s welfare; not merely avoid harmful acts. Beneficence has a connection to mercy, kindness, and charity and includes altruism, love, and, as important in the case of non-human animals, humanity. The principle means there is a moral obligation to act for the benefit of others [34]. Here motives and action are important. From a human bioethical perspective, the principle of positive beneficence embraces moral rules of obligation and include: (1) protecting and defending the rights of others (in this paper’s perspective, non-human moral subjects), (2) preventing harm from occurring to others (required action to object to or prevent deliberate or even unintentional harm to the moral subject), (3) removing conditions that will cause harm to others (providing optimal husbandry laboratory conditions for both research organisms and aquaculture operations including space, water quality, oxygen, proper temperatures and lighting, etc.), (4) helping persons with disabilities (not applicable in this context) and rescue persons in danger (taking steps to immediately remove or rectify conditions wherein the well-being of the moral subject is being compromised) [34].

The difference between nonmaleficence and beneficence parallels the difference between consequentialist ethics application (one of utility or seeking the best good) and a deontological ethics where the primary function is duty to others. Many deontological ethical constructs are negative in approach such as do no harm. It has fewer duties to actually be focused on the positive. Thus, deontological ethics promotes nonmaleficence over beneficence [41]. In contrast, consequential ethical constructs focus on the idea one should always strive or follow the rule that leads to the best consequences. In essence, doing good is better than neither doing good nor bad. From a consequentialist perspective, one is just as culpable in failing to prevent harm as they would be in actually causing harm. Consequentialist ethics promotes not only nonmaleficence but also beneficence [41].

From the context of marine biotechnology research, development, and aquaculture, a beneficence principle means the researcher or organization must take proactive steps to ensure their actions yield positive results for humans, but for non-humans as well. This effort may present an ethical dilemma as often both cannot be accomplished. For such consideration, we look to our fourth bioethical moral principle.

### 2.6. Justice

The basis for justice with regard to bioethics originates conceptually with Aristotle with the context of “equals must be treated equally and unequals must be treated unequally [34]. Under this principle there is no argument with regard to equals, but how does one apply such “formal justice” to unequals where differences (e.g., human versus non-human) are so relevant [34]? Here it becomes a question of how far should equality extend. 

This concept was codified by John Rawls in 1971 with his *Theory of Justice* [45] that led more to a political theory than an ethical normative theory. Although Rawls himself really did not address animal well-being in his theory, it has since taken on a life of its own and has a devout following; especially among more liberal ethicists and philosophers [46]. It also has since gained considerable traction among some segments of the general public.

Herein lies a consideration issue of this commentary’s goal as there are many passionate individuals who fully believe that non-human animals in research and/or food-production operations deserve or are entitled to the same rights as their conspecifics from nature. To that end, animal rights groups often demand that animal research and production facilities adhere to fairness and justice with regard to how the animals are treated. Adherents on the extreme side would shut down all animal research and/or food-production facilities. In fairness, individuals or groups professing this philosophy may not be so much for justice as they are against injustice [41]. As one would expect, there are many nuanced forms of justice, each with its own sets of rules and applications, and each links to a different form of ethical theory.

For a perspective of just experimentation under the auspices of biotechnology, one is referred to Stephen Clark’s discussion on the theory of just experimentation [47]. Interestingly, Clark states that some researchers espouse animals that have no moral weight at all thus rendering the question of moral consideration moot. Others have argued the opposite—no invasive experimentation is justified under any circumstance and no sentient animal should be exploited as a means of profit for another “superior class of individuals” [47]. 

There are also concerns of intrinsic wrongs that would fall under the guise of justice. For instance, the concept of an organism’s *telos* (purpose or design in life) is not to be harmed or hindered. This would mean that genetic manipulation could compromise the integrity of an organism. Such questions are framed philosophically under the question of what constitutes a being, and do we have a right to change that individual’s intrinsic right to exist and meet its life purpose? For example, would genetic manipulation impact reproductive success and hence fitness if the intent is to produce a sterile organism (i.e., a triploid organism) that shunts all energetic intake into growth instead of partitioning between growth and reproduction (as a normal diploid individual). Does such action violate an organism’s *telos*? Understandably this is a philosophical argument. Yet, from a public perspective such manipulation may be problematic as they may perceive the end game only focusing on maximizing profit and not the overall organism’s well-being. Alternatively, one could question the validity of public concern over profit consideration while the purpose of the manipulation is designed to minimize potential genetic contamination if the organism escaped from a research or production facility into the wild. Consideration of and answers to such concerns could mitigate public outcry.

Clark listed the British Farm and Animal Welfare Council’s intrinsically objectionable issues as: “(1) inflicting very severe or lasting pain on the animals concerned, (2) violating the integrity of a living being (the *telos* question), (3) mixing two kinds to ‘an extent which is unacceptable’; and (4) generating living beings whose sentience has been reduced to the extent that they may be considered mere instruments or artifacts” [47]. He went on to present counter arguments with supported discussion as to why these are not *absolute* wrongs but potentially more of *intrinsically undesirable acts* that may be excused under certain circumstances. 

One must consider that the benefits of technology are, in principle, geared toward improvement in health and safety of humans. Likewise, from a global seafood consumption perspective, marine biotechnology will serve to only improve the efficiency of food production under confined conditions. Concomitantly, researchers and farm managers are consciously and constantly taking appropriate steps to ensure that the organisms being cultured are cared for with as much consideration to their overall welfare as possible. To do otherwise would not make scientific nor economic sense.

## 3. The Cost of Inappropriate Action

Marine biotechnology and aquaculture research and development, regardless of the purpose, are coming under growing scrutiny by a more-informed public. Scientists and producers must not let the technology get ahead of the implications and knowledge of short-term impacts of such research. Likewise, we should be constantly striving to understand the long-term effects on the organism, its genetic and/or *telos* integrity, the environment in which it lives, and the protection of the integrity of science itself. 

Personally, I serve on our Institutional Animal Care and Use Committee as an aquatic scientist and as an ethicist. I also serve as our college’s Research Integrity Officer who investigates scientific misconduct among faculty and our partners. The amount of public scrutiny that I have observed over the past few years, and the concerns of both undergraduates and graduate students about animal welfare and scientific integrity is growing. The impact of these concerns could directly influence the funding agencies who are willing to support our research efforts because misconduct or unethical treatment of animals has bottom-line implications for the agency as well as the researcher. Government agencies are being held to a greater standard of accountability as to how public funds are spent. All it takes is a few mistakes regardless of intentionality that make the front page of local, state, or national news to result in a shutdown of research-funding priorities. Likewise, from a business ethic perspective all one has to do is look at the company’s balance sheet and recognize that “Good Will” is an asset. Any company that compromises the public trust by violating its social contract will see the result reflected in their Good Will asset. Stakeholder wealth will decline and the company leadership will be in jeopardy.

These admonitions lead one to consider that, because the marine biotechnology industry and aquaculture are not mature industries, is it now the time to consider the ethical implications of one’s research and/or production. It is important to remember that a basic tenant of ethics is not “can we do something, but should we.” Science continues to have great promise and the technological advances that come from its findings have significant implications to the health, well-being, and success of humans on a global scale. We must not forget that the whole movement in animal ethics began when there was a legitimate antivivisectionist’s outcry in the 1800s and science ignored the concerns. As the marine biotechnology science and aquaculture industries begin to be mature, we need to be proactive instead of reactive to public outcries against inhumane treatment of non-human animals, even if there is a question of sentience or cognitive understanding. 

## 4. Intermediate and Alternative Options

I propose that those individuals directly involved in marine biotechnology research and aquaculture production form a working group that examines the bioethical implications of their science and application using the principles of medical bioethics as a starting point. This working group should include scientists, biologists, ethicists, regulatory personnel, religious representatives, and the general public. One must also recognize the limitations of the principlists’ approach because its procedural tenants do not conform to non-human animals. However, it is a good starting point. 

Thoughtful discussion will have to come into play as to what one should consider warrants moral considerability. This consideration should be reconciled with what is published in the scientific literature, by listening to and finding common ground with the various animal ethics and religious groups, and basing it on what we currently know from a physiological, behavioral, and cognitive ability of the “moral subject” in question. As the moral agent, it is the responsibility of scientists and producers to be aware of and take appropriate steps to take into account the welfare or welfare interests of the subject. To do so means that one must recognize and develop an ethic that parallels accepted norms but has application for many subjects that do not fit within that norm. In other words, where and when is it appropriate to bring the animals (subjects) into the realm of moral considerability? 

Alternatively, one could consider if you can justify why such an organism would be exempt from such consideration. In this situation, if you categorically and justifiably place an organism outside moral consideration, the bioethical principles do not apply. If this is the case, to be proactive to avoid potential conflict from disagreements, one may want to consider a few of the more applicable recommendations by Clark [47]. He recommends avoiding the following;

“…, 2) the creation of human—animal hybrids; 3) any work that can be expected to produce creatures who would suffer severe or lasting distress (including animals to be created as disease models unless there is clear evidence that the problems can be handled humanely); 4) the production at least of chimeras and those hybrids which involve hybridisation outside closely related taxa; … 6) any work intended to strip organisms of their species-specific biograms (*telos*), or render them incurably insentient; 7) any *unguided* genetic modification that runs ahead of reasonable guesses about the function of particular genes, and the chance of managing any damaging effects”.

[47]

Given the complexities of non-human animals fitting within a principlist paradigm, another alternative may be to consider a balance of bioethics and environmental stewardship. In this scenario, ethical consideration is linked directly to the intrinsic nature of the subject by a concept of sustainability protecting wild organisms while cultivating domesticated or manipulated organisms that will not escape into the wild and are treated in the same manner as other agricultural livestock or products. This approach should aid in removing the utility aspect as the good of the animal is balanced between human needs, the organism’s welfare, and wild-stock and environmental protection. Here autonomy is not an issue per se because the focus is on sustainability of the whole rather than the individual. Likewise, nonmaleficence and beneficence are inherently considered because the goal is to maximize growth conditions and the organism’s well-being. Lastly, such a balanced model would address justice concerns as all organisms are being treated equal and none are being taken from the wild once founder stocks are fully developed and the life-cycle of the organism is closed. The ultimate key here is to avoid gratuitous evil or the appearance of gratuitous evil where harm and lack of beneficence is being conducted. In considering this alternative model, one addresses not only the welfare of the organisms in question but also takes initiative to protect the environment and address the intrinsic (not instrumental) value of the organism.

## References

[1] Bocquet-Appel J.P. (2011). When the world’s population took off: The springboard of the Neolithic demographic transition. Science.

[2] Chassy B.M., Parrott W.A., Roush R. (2005). Crop Biotechnology and the Future of Food: A Scientific Assessment.

[3] Travis J. (2015). Making the cut. Science.

[4] Mitchell C.B., Pellegrino E.D., Elshtain J.B., Kilner J.F., Rae S.B. (2007). Biotechnology and the Human Good.

[5] Matthews K.S., Rudolph F.B., McIntire L. (1996). Overview of terminology and advances in biotechnology. Biotechnology: Science, Engineering and Ethical Challenges for the 21st Century.

[6] Grigorakis K. (2010). Ethical issues in aquaculture production. J. Agric. Environ. Ethics.

[7] Schnieke A., Englehard M., Hagen K., Boysen M. (2009). Animal pharming: Past experience and future prospects. Genetic Engineering in Livestock: New Applications and Interdisciplinary Perspective.

[8] Wall R., Laible G., Maga E., Seidel G., Whitelaw B. (2009). Animal Productivity and Genetic Diversity: Cloned and Transgenic Animals.

[9] Rehbinder E., Engelhard M., Hagen K., Jørgensen R.B., Pardo-Avellaneda R., Schnieke A., Thiele F. (2010). Pharming: Promises and Risks of Biopharmaceuticals Derived from Genetically Modified Plants and Animals.

[10] Myhr A.I., Dalmo R.A. (2005). Introduction of genetic engineering in aquaculture: Ecological and ethical implications for science and governance. Aquaculture.

[11] Keefer C.L., Pommer J., Robl J.M. (2007). The Role of Transgenic Livestock in the Treatment of Human Disease.

[12] Niemann H., Kues W., Carnwath J.W., Englehard M., Hagen K., Boysen M. (2009). Transgenic farm animals: Current status and perspectives for agriculture and biomedicine. Genetic Engineering in Livestock: New Applications and Interdisciplinary Perspective.

[13] Thompson P.B., Blazer F.W., Einsiedel E. (2010). Ethical Implications of Animal Biotechnology: Considerations for Animal Welfare Decision Making.

[14] Frewer L., Lassen J., Kettliz B., Scholderer J., Beekham V., Berdal K.G. (2004). Societal aspects of genetically modified foods. Food Chem. Toxicol..

[15] Slovic P. (1993). Perceived risk, trust and democracy. Risk Anal..

[16] Matthias K., Creswell R.L., Flos E. (2002). Social perceptions and ethics in aquaculture: Aquaculture as a responsible supplier for the new millennium. Perspectives on Responsible Aquaculture for the New Millennium.

[17] Bovenkerk B., Meijboom F.L.B. (2012). The moral status of fish. The importance and limitations of a fundamental discussion for practical ethical questions in fish farming. J. Agric. Environ. Ethics.

[18] Bovenkerk B., Meijboom F.L.B. (2013). Fish welfare in aquaculture: Explicating the chain of interactions between science and ethics. J. Agric. Environ. Ethics.

[19] Berqvist J., Gunnarsson S. (2013). Finfish aquaculture: Animal welfare, the environment, and ethical implications. J. Agric. Environ. Ethics.

[20] Millar K., Tomkins S. (2007). Ethical analysis of the use of GM fish: Emerging issues for aquaculture development. J. Agric. Environ. Ethics.

[21] Pimentel D., Shanks R.R., Rylander J.C. (1996). Bioethics of fish production: Energy and the environment. J. Agric. Environ. Ethics.

[22] Food and Agriculture Organization of the United Nations, Rome (FAO) (2005). Ethical Issues in Fisheries.

[23] Hew C.L., Fletcher G.L. (2001). The role of aquatic biotechnology in aquaculture. Aquaculture.

[24] Tacon A. (2004). Contribution of aquaculture to global food supply: An overview. Aquat. Resour. Cult. Dev..

[25] Food and Agriculture Organization of the United Nations, Rome (FAO) (2006). The State of World Fisheries and Aquaculture 2006.

[26] Food and Agriculture Organization of the United Nations, Rome (FAO) (2016). The State of World Fisheries and Aquaculture 2016.

[27] Grundwald D.J., Eisen J.S. (2002). Headwaters of the zebrafish—Emergence of a new model vertebrate. Nat. Rev. Genet..

[28] Udvadia A.J., Linney E. (2003). Windows into development: Historic, current, and future perspectives on transgenic zebrafish. Dev. Biol..

[29] Beermann D.H., Dunshea F.R. (2005). Metabolic Modifiers for Use in Animal Production.

[30] Naylor R.L., Goldburg R.J., Priavera J.H., Kautsky N., Beverigde M.C.M., Clay J., Folke L., Lubchenco J., Mooney H., Yroell M. (2000). Effect of aquaculture on world fish supplies. Nature.

[31] Sagar A., Daemmrich A., Ashiya M. (2000). The tragedy of the commoners: Biotechnology and its public. Nat. Biotechnol..

[32] Wynne B. (1992). Uncertainty and environmental learning: Reconceiving science and policy in the preventative paradigm. Glob. Environ. Chang..

[33] National Research Council (NRC) (2002). Animal Biotechnology: Science Based Concerns.

[34] Beauchamp T.L., Childress J.F. (1979). Principles of Biomedical Ethics.

[35] Matthias K., Forsberg E.-M. (2001). Assessing Fisheries—Using an ethical matrix in a participatory process. J. Agric. Environ. Ethics.

[36] Matthias K., Millar K., Thorstensen E., Tompkins S. (2007). Developing the ethical matrix as a decision support framework: GM fish as a case study. J. Agric. Environ. Ethics.

[37] Lam M.E. (2016). The ethics and sustainability of capture fisheries and aquaculture. J. Agric. Environ. Ethics.

[38] Platt J.L., Prather R.S. (2004). Animal Organ Donors: Human Health Applications.

[39] Turner L. (2009). Does bioethics exist?. J. Med. Ethics.

[40] Potter V.R. (1996). Real bioethics: Biocentric or anthropocentric?. Ethics Environ..

[41] Baggini J., Fosl P.S. (2012). The Ethics Toolkit: A Compendium of Ethical Concepts and Ethods.

[42] Singer P.F. (2009). Animal Liberation: The Definitive Classic of the Animal Movement.

[43] Regan T. (1983). The Case for Animal Rights.

[44] Thomas N. (2016). Animal Ethics and the Autonomous Animal Self.

[45] Rawls J. (1971). A Theory of Justice.

[46] Abbey R. (2007). Rawlsian resources for animal ethics. Ethics Environ..

[47] Clark S.R.L., Deane-Drummond C., Szerszynksi B. (2003). Thinking about biotechnology: Towards a theory of just experimentation. Reordering Nature: Theology, Society, and the New Genetics.

